# Combination of deep learning and ensemble machine learning using intraoperative video images strongly predicts recovery of urinary continence after robot‐assisted radical prostatectomy

**DOI:** 10.1002/cnr2.1861

**Published:** 2023-07-14

**Authors:** Wataru Nakamura, Makoto Sumitomo, Kenji Zennami, Masashi Takenaka, Manabu Ichino, Kiyoshi Takahara, Atsushi Teramoto, Ryoichi Shiroki

**Affiliations:** ^1^ Department of Urology, School of Medicine Fujita Health University Toyoake Japan; ^2^ Fujita Cancer Center Fujita Health University Toyoake Japan; ^3^ Faculty of Radiological Technology, School of Medical Sciences Fujita Health University Toyoake Japan; ^4^ Faculty of Information Engineering Meijo University Nagoya Japan

**Keywords:** deep learning, ensemble machine learning, robot‐assisted radical prostatectomy, urinary continence, video images

## Abstract

**Background:**

We recently reported the importance of deep learning (DL) of pelvic magnetic resonance imaging in predicting the degree of urinary incontinence (UI) following robot‐assisted radical prostatectomy (RARP). However, our results were limited because the prediction accuracy was approximately 70%.

**Aim:**

To develop a more precise prediction model that can inform patients about UI recovery post‐RARP surgery using a DL model based on intraoperative video images.

**Methods and Results:**

The study cohort comprised of 101 patients with localized prostate cancer undergoing RARP. Three snapshots from intraoperative video recordings showing the pelvic cavity (prior to bladder neck incision, immediately following prostate removal, and after vesicourethral anastomosis) were evaluated, including pre‐ and intraoperative parameters. We evaluated the DL model plus simple or ensemble machine learning (ML), and the area under the receiver operating characteristic curve (AUC) was analyzed through sensitivity and specificity. Of 101, 64 and 37 patients demonstrated “early continence (using 0 or 1 safety pad at 3 months post‐RARP)” and “late continence (others),” respectively, at 3 months postoperatively. The combination of DL and simple ML using intraoperative video snapshots with clinicopathological parameters had a notably high performance (AUC, 0.683–0.749) to predict early recovery from UI after surgery. Furthermore, combining DL with ensemble artificial neural network using intraoperative video snapshots had the highest performance (AUC, 0.882; sensitivity, 92.2%; specificity, 78.4%; overall accuracy, 85.3%) to predict early recovery from post‐RARP incontinence, with similar results by internal validation. The addition of clinicopathological parameters showed no additive effects for each analysis using DL, EL and simple ML.

**Conclusion:**

Our findings suggest that the DL algorithm with intraoperative video imaging is a reliable method for informing patients about the severity of their recovery from UI after RARP, although it is not clear if our methods are reproducible for predicting long‐term UI and pad‐free continence.

## 1 INTRODUCTION

Artificial intelligence (AI) is a computer science that approximates human cognitive functions such as decision‐making, problem‐solving, detection, and classification using algorithms.^1^ A new machine learning (ML) technology called deep learning (DL)^2^ is in increasing demand in the medical field.^3^, ^4^, ^5^ Urology was one of the first areas where AI was used for detecting medical devices, identifying images, assessing surgical skill, and predicting clinical effectiveness in complex urological procedures.^6^

Prostate cancer (PC) affects a high percentage of men worldwide, and robotic‐assisted radical prostatectomy (RARP) is the standard of care for localized PC. However, post‐prostatectomy urinary incontinence (PPUI) is a typical postoperative complication that significantly impairs the quality of life of those with PC. We recently reported that DL with magnetic resonance imaging (MRI) is useful for predicting the UI severity after RARP.^7^ Our results suggested that a DL algorithm using preoperative imaging might aid treatment selection, especially for PC patients who wish to avoid long‐term UI after RARP. However, results were limited, as the accuracy of prediction was only about 70%.^7^ Recent studies suggest that the preservation of periprostatic structures by intraoperative surgical techniques such as nerve‐sparing (NS), bladder neck‐preserving, and Retzius‐sparing modalities are associated with early recovery from UI after RARP.^8^, ^9^, ^10^ Although surgical procedures can be expected to improve early recovery from UI, it is difficult to objectively assess the relationship between surgical procedures and the preservation of anatomic structures and whether they contribute to early recovery from UI after RARP.

We hypothesized that DL could objectively assess the relationship between surgical techniques and the preservation of anatomic structures and the relationship between these anatomical structures and early recovery from UI after RARP. In the present study, we aimed to develop a more accurate predictive model to inform patients with PC about the timing of UI recovery after RARP surgery, using a DL model based on intraoperative video images.

## 2 MATERIALS AND METHODS

### 2.1 Patients selection

All experimental protocols were approved by the Institutional Review Board (IRB) of Fujita Health University School of Medicine (IRB no. HM19‐257). All methods were performed in accordance with the relevant local guidelines and regulations. The patients were explained the purpose of the study, and a website with additional information, including an opt‐out option, was set up for the study. A database of 400 patients with PC was used in our recent study (from August 1, 2015 to July 31, 2019).^7^ In 78 patients, surgical videos had been deleted or lost, and in 299 patients, video records could be viewed; however, snapshots necessary to perform the analysis could not be obtained (refer to Snapshots extraction from the intraoperative video). Thus, we included 101 patients whose intraoperative video records were available. In addition, the video records of 30 additional patients undergoing surgery were prepared for internal validation.

### 2.2 RARP surgery

RARP was performed by nine surgeons using the da Vinci Si or Xi system (Intuitive Surgical, Inc., Sunnyvale, CA, USA). NS surgery was performed according to the clinical stage and risk criteria, and bladder neck preservation was routinely included. Every patient underwent posterior and anterior reconstruction.

### 2.3 Pre‐ and intraoperative risk parameters and the continence definition

Preoperative clinicopathological covariates, such as age, body mass index (BMI), neoadjuvant androgen deprivation therapy (NADT) history, membranous urethral length (MUL), prostate volume (PV), continence status before RARP, serum prostate‐specific antigen (PSA) level, Gleason score (GS sum), clinical stage, and risk criteria based on the risk stratification in the European Association of Urology guidelines, and intraoperative covariates, such as operator experience, total operation time, console time, with or without NS, and bleeding volume, were assessed. We considered surgeons with more than 50 cases of RARP surgery experience as experts, whereas the others were non‐expert. Continence was evaluated using the Expanded Prostate Cancer Index Composite survey question: “How many pads per day did you usually use to control leakage during the last 4 weeks?” Patients who did not use pads with no urine leakage or used 1 safety pad for less than 20 mL at 3 months postoperatively were included in the “early continence” group, whereas others were categorized into the “late continence” group.

### 2.4 Snapshots extraction from the intraoperative video

**FIGURE 1:**
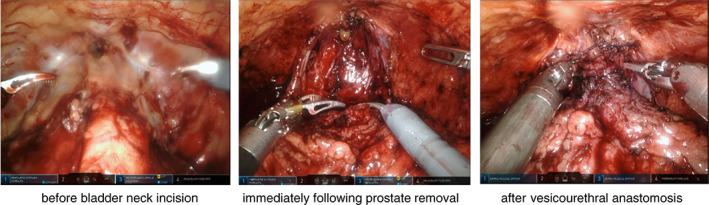
Representation of the three snapshots extracted from the intraoperative video of the same patients. Three snapshots showing the pelvic cavity (before bladder neck incision, immediately after prostate removal, and after vesicourethral anastomosis) from the intraoperative video records. Snapshot extraction was performed as described in Section 2.

Three snapshots from intraoperative video recordings showing the pelvic cavity (prior to bladder neck incision, immediately following prostate removal, and after vesicourethral anastomosis) were extracted. Snapshot extraction was performed in accordance with the following principles while considering reproducibility: (1) Anatomical structures near the pubic symphysis (prostatic apex) should be included in all images. (2) In “before bladder neck incision,” the bladder neck and prostatic apex should be visible. (3) In the “immediately after prostate removal,” bladder neck preservation, nerve preservation, and degree of bleeding should be visible. (4) In “after vesicourethral anastomosis,” the anastomosis should be visible without tension on the urethra or bladder. Figure 1 shows the representation of the three snapshots extracted from the intraoperative video records of the same patients.

### 2.5 DL model

First, the given images were input into a convolutional neural network (CNN), which is a DL technique^2^, ^11^ that has an excellent ability to classify images.^12^ CNN is applied in medical image processing and is widely used for lesion detection and differentiation and prognostication.^7^, ^13^, ^14^ We focused on DenseNet, which is a type of CNN.^15^ By tightly coupling the layers, information can be transmitted smoothly, even in a multilayered network, thereby improving the processing performance. DenseNet has several variations with different numbers of network layers; in this study, we used DenseNet169 (with 169 layers).

A common method for using CNNs is to input data and directly obtain the desired results, such as the classification output. In this study, three images were treated as input images; therefore, a simple DenseNet could not be used. CNNs are also used as feature extractors, where the convolutional layer of the CNN is responsible for extracting various features from the input images. CNNs trained on a large number of images can extract general‐purpose features from images, and some studies have used these features for other purposes. In our previous study,^7^ a CNN was used to extract 4096 features from a single image and predict whether urinary continence was good or poor using ML.

In this study, three images were input into DenseNet169, and 1920 features were obtained for each image. Overall, 5760 features were used for prediction. Dimensionality compression was required because the quantity of features was large in comparison to the number of samples. Principal component analysis was used for dimensionality compression, and the data were compressed into 20‐dimensional principal components. These data were the image features obtained from the CNN.

Using the image features and clinical information obtained as described above, an ML method was used to predict whether urinary continence was early or late. Naïve Bayes, support vector machine, random forest, and artificial neural networks (ANNs) were each used as the ML method. This method of prediction, using only one ML method, is called the single model. We also introduced an ensemble model, in which the output of the above four ML methods (probability of UI) and 20 compressed features were input again to ML to predict UI.

**FIGURE 2:**
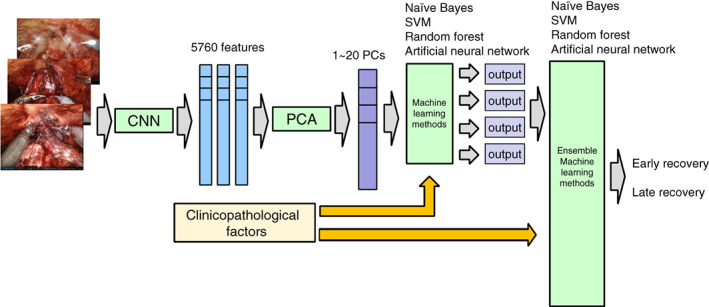
Automated classification method of the early recovery of urinary continence using DL and simple ML or DL and ensemble ML. Intraoperative video images were input to pretrained VGG‐16, and 20 kinds of features that contribute to classification were selected using information obtained from 5760 output values of the last convolutional layer extracted as characteristic features. Subsequently, the selected image features and preoperative and intraoperative parameters were given to a plurality of ML algorithms to distinguish between good and bad urinary incontinence. Ensemble ML was performed instead of simple ML. DL, deep learning; ML, machine learning; VGG‐16, Visual Geometry Group 16.

The following six methods (Methods 1–6) were possible depending on the variation in input information (images and clinical information) and network configuration (simple and ensemble models). In this study, we compared the prediction performances of these methods as follows: Method 1, DL with three video images and simple ML; Method 2, DL with three video images and ensemble ML; Method 3, DL with 3 video images, addition of clinical factors, and simple ML; Method 4, DL with three video images, addition of clinical factors, and ensemble ML; Method 5, simple ML using clinicopathological factors; and Method 6, ensemble ML using clinicopathological factors. These DL methods with simple and ensemble ML classification models for postoperative UI recovery are summarized in Figure 2.

### 2.6 Statistical analyses

The EZR software (Saitama Medical Center, Jichi Medical University, Saitama, Japan)^16^ was used for statistical analyses. Data between the early continence and the late continence groups were compared using the Mann–Whitney test and Fisher's analysis. Multivariate logistic regression analyses were performed to evaluate the variables associated with postoperative continence. Statistical significance was defined as *p* < 0.05.

## 3 RESULTS

The median age and BMI at the time of RARP among the 101 patients were 66 years and 23.4 kg/m^2^, respectively. The median PSA level and PV at PC diagnosis were 7.50 ng/mL and 27.2 mL, respectively. The GS sum obtained by prostate biopsy was 6 in 28 (28%), 7 in 52 (51%), and >8 in 21 (21%) patients. The risk criteria were low in 17 (16.8%), intermediate in 49 (48.5%), and high in 35 (34.7%) patients. Twenty‐seven (26.7%) patients had received NADT. Sixty‐two patients were operated by skilled expert surgeons, while 39 patients were operated by unskilled non‐expert surgeons. The median operative time, console time, and bleeding volume were 160 min, 119 min, and 200 mL, respectively. NS surgery was performed in 80 patients (unilateral, *n* = 70; bilateral, *n* = 10). A histopathologically positive resection margin was observed in 19 patients (18.5%).

The characteristics of the early continence group (64 patients) and late continence group (37 patients) at the 3‐month follow‐up are presented in Table 1. There were significant differences between groups with regard to the median BMI, GS sum, and MUL, as well as NADT history. Although no significant differences in intraoperative parameters were observed, there was a tendency for NS surgery to affect continence. The logistic regression analyses to evaluate continence status at 3 months postoperatively showed that BMI, NADT history, MUL, and NS remained significant predictors of poor continence (Table 2); these findings were notably similar to our previous study.^7^

**TABLE 1:** Comparison of characteristics between early and late continence groups 3 months after RARP.

	Early continence group (*n* = 64)	Late continence group (*n* = 37)	*p*‐Value
Median age, years (IQR, range)	65.5 (60–69, 45–75)	66 (62–69, 49–76)	0.589
Median BMI, kg/m^2^ (IQR, range)	22.9 (21.7–24.1, 18.3–28.7)	24.4 (21.5–26.0, 17.9–30.1)	0.028
NADT			
No	56	18	<0.001
Yes	8	19	
PV, cm^3^ (IQR, range)	27 (22–36, 14–77)	27 (20–40, 13–93)	0.781
MUL, mm (IQR, range)	13 (11–14, 8–17)	12 (11–13, 7–16)	0.011
Incontinent before RARP			
No	64	36	0.366
Yes	0	1	
PSA, ng/mL (IQR, range)	6.90 (5.19–10.0, 3.2–24.0)	8.41 (5.90–11.9, 3.2–28.6)	0.107
GS sum (IQR, range)	7 (7–8, 6–9)	7 (7–7, 6–9)	0.008
*T* stage			
≦2*b*	53	26	0.222
≧2*c*	11	11	
Risk criteria			
Low	12	5	0.083
Intermediate	35	14	
High	17	18	
Operated by			
Expert surgeons	35	27	0.108
Non‐expert surgeons	29	10	
Operation time (IQR, range)	164 (129–195, 90–267)	155 (141–180, 107–270)	0.501
Console time (IQR, range)	125 (90–153, 59–220)	110 (98–126, 77–230)	0.327
Bleeding volume (IQR, range)	112 (50–200, 30–600)	100 (60–150, 10–400)	0.397
Nerve sparing not done	10	11	0.065
Unilateral	45	25	
Bilateral	9	1	

**TABLE 2:** Logistic regression analyses of predictive factors, including intraoperative factors on PPUI 3 months after RARP.

	Odds ratio	95% CI	*p*‐Value
Age	1.000	0.90–1.10	0.983
BMI	1.160	1.05–1.44	**0.003**
NADT	14.60	2.50–82.2	**0.023**
PV	1.020	0.98–1.07	0.222
MUL	0.905	0.66–0.97	**0.002**
Continence status before RARP	1.245	0.40–5.85	0.578
PSA	1.030	0.90–1.16	0.671
GS sum	1.04	0.39–2.76	0.930
T stage ≧ 2*c* (vs. ≦2*b*)	1.31	0.20–8.40	0.774
Risk criteria	2.26	0.25–19.8	0.461
High (vs. low or intermediate)			
Non‐expert surgeon	0.293	0.06–1.38	0.121
Operation time	1.040	0.98–1.10	0.121
Console time	0.958	0.90–1.02	0.177
Bleeding volume	1.000	0.99–1.00	0.971
Nerve sparing	0.328	0.24–0.88	**0.034**

*Note*: *p*‐values less than 0.05 with statistically significant differences are bolded.

**FIGURE 3:**
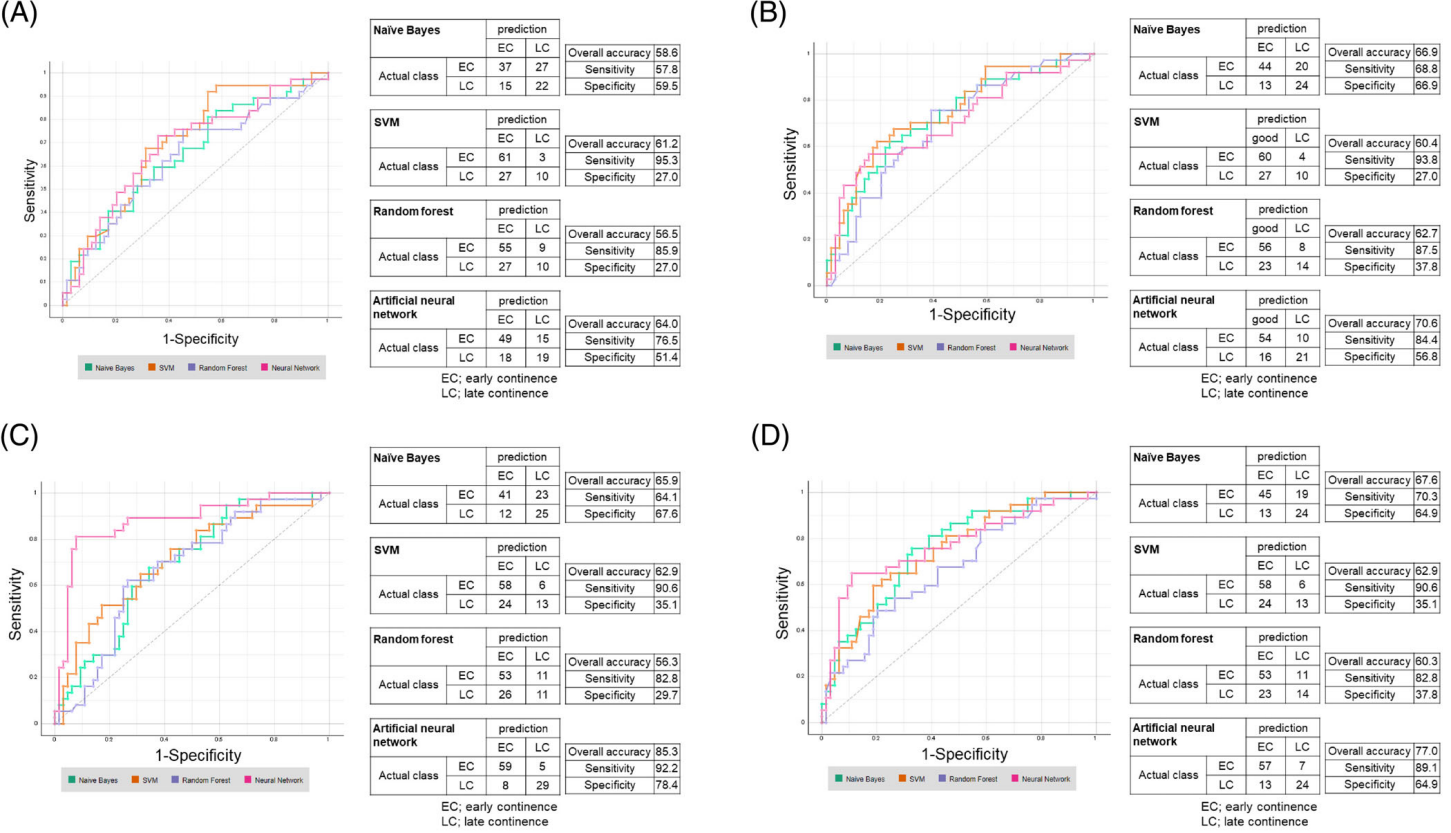
ROC curves and accuracies on continence prediction using intraoperative video images and clinicopathological parameters analyzed by DL and simple ML or DL and ensemble ML. (A, B) Intraoperative video images with (A) or without (B) clinicopathological parameters were analyzed by DL and simple ML, as described in Figure 1. ROC analyses were performed three times, and the representatives were shown. (C, D) Intraoperative video images without (C) or with (D) clinicopathological parameters were analyzed by DL and ensemble ML, as described in Figure 1. ROC analyses were performed three times, and the representatives were shown. DL, deep learning; ML, machine learning; ROC, receiver operating characteristic.

**FIGURE 4:**
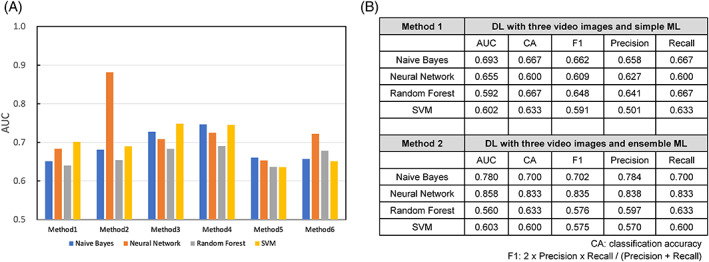
AUCs and accuracies according to six kinds of methods in the 101 patient data set and internal validation set. Intraoperative video images with or without clinicopathological parameters were analyzed by DL and simple or ensemble ML, as described in the Materials and Methods. ROC analyses were performed, and AUCs were calculated. (A) AUCs according to six kinds of methods in the 101 patient data set are shown. (B) An internal validation test was performed using snapshot photographs extracted from surgical videos of 30 recently operated patients. AUCs and accuracies according to Methods 1 and 2 are shown. Each analysis was performed three times with similar results, and the representatives were shown. AUC, area under the receiver operating characteristic curve; DL, deep learning; ML, machine learning; ROC, receiver operating characteristic.

Figure 3A,B shows the AUC and the accuracy of continence prediction using Methods 1 and 3. Those results showed that combining DL with simple ML using intraoperative video snapshots with clinicopathological parameters (Methods 3) demonstrated a higher performance (AUC, 0.683–0.749) for predicting early recovery from PPUI (Figure 3A and Table S1), while intraoperative video snapshots alone (Method 1) achieved an AUC of 0.641–0.701 (Figure 3B and Table S1), suggesting that the combination of intraoperative video images with clinicopathological parameters showed additive effects for PPUI prediction. We then attempted a combination of DL and ensemble ML using intraoperative video images (Methods 2 and 4). We found that combining DL with ensemble ANN using intraoperative video snapshots (Method 2) had the highest performance, with an AUC of 0.882 (sensitivity, 92.2%; specificity, 78.4%; overall accuracy, 85.3%) for predicting early recovery from PPUI (Figure 3C and Table S1). In contrast, DL with three video images, addition of clinical factors, and ensemble ML (Method 4) achieved no additive effects (AUC, 0.690–0.747) compared with DL and simple ML with clinicopathological parameters (Figure 3D and Table S1). The AUCs of the four ML algorithms according to the DL and ML methods are shown in Figure 4A. Ensemble ML involving clinicopathological parameters had a non‐notable additive effect on performance compared with simple ML (Methods 5 and 6 in Figure 3). We finally performed an internal validation test using snapshot photographs extracted from surgical videos of 30 recently operated patients. Although the combination of DL and simple ML using intraoperative video snapshots (Methods 1) did not give excellent results, the combination of DL and ensemble ANN (Method 2) performed best in predicting early recovery from PPUI with 0.858 AUC (Figure 4B). These results suggest that ensemble ML has the potential to improve accuracy using information from intraoperative video images, but not with clinicopathological parameters, including intraoperative parameters.

## 4 DISCUSSION

Many researchers have conducted studies to determine preoperative and intraoperative parameters^17^, ^18^, ^19^ that may be useful to identify patients at risk of PPUI. Recent studies have shown a correlation between good surgeon skill and excellent continence outcomes after RARP,^20^, ^21^, ^22^ which has been proven by AI methods using semantic segmentation and automated performance status (APM). These findings strongly suggest that PPUI is influenced by surgeon (technical) and patient (anatomical) factors. In our previous study,^7^ we reported that the overall accuracy for predicting early recovery from PPUI was at most 70% using a DL model based on preoperative pelvic MRI. Hung et al. also reported that the overall accuracy was approximately 70% using the surgeon APM and clinical parameters.^21^ In the present study, our DL model combined with simple ML using intraoperative video images produced similar results (AUC, approximately 0.65–0.70) for predicting early recovery from PPUI. We then applied ensemble methods to classify PPUI status. The ensemble method is an ML technique that combines multiple base models to create an optimal predictive model, and it has been used in recent studies.^23^, ^24^ Notably, the DL model combined with the ensemble ANN showed over 80% accuracy in predicting good recovery from PPUI. To our knowledge, this study is the first to report that a DL model using intraoperative video snapshots can improve the accuracy of early recovery from UI after RARP surgery compared to previous reports on PPUI. Moreover, this is the first study to predict postoperative outcomes (including prognosis and complications) via DL using intraoperative video images rather than APM or semantic segmentation.

Our model was able to predict the risk of PPUI with various ML models using preoperative and/or intraoperative factors. Each ML method has its advantages and disadvantages, and it is necessary to determine a suitable model for classification by experimenting with various methods. Support vector machines, random forests, and naïve bays place data in a multidimensional space and classify them using hyperplanes based on a policy of minimizing structural risk.^25^, ^26^ On the other hand, ANN is uniquely aimed at solving problems that arise during various classifications and pattern recognition.^27^ ANN was developed to mimic the neuronal ecology of the human brain and is trained to reflect weighted combinations of input variables in its results.^28^, ^29^ The greatest advantage of ANN is its ability to efficiently approximate and analyze any nonlinear functional model.^26^ Based on the above, it is plausible that the AUC improves dramatically only when DL and ensemble ANN are used, as noted in the results of this study. However, it is difficult to identify what DL combined with ensemble ANN focuses on because of the following reasons. First, as each of the 20 factors obtained by PCA integrates multiple characteristics, it is challenging to analyze the importance of individual factors. Second, multiple video snapshots made using gradient‐weighted class activation mapping impossible; this is known as the black‐box problem.^30^ Focusing on previously reported PPUI‐related factors may shed some light on why DL and ensemble ANNs improved AUC. Although many reports, including this study, have shown that MUL is one of the preoperative predictors of PPUI,^7^, ^31^, ^32^, ^33^ we doubt that MUL specificity alone can explain the prediction using the DL and ensemble ANN. This is because the addition of clinicopathological parameters including MUL failed to show additive effects for each analysis using DL, EL and simple ML. Recent studies have reported the importance of anatomical structure such as urethral wall thickness^34^ and levator ani muscle thickness^35^ as well as previously reported MUL as urethra‐related factors for PPUI in prostatectomy. It might be hypothesized that the ANN method is capable of identifying differences in sphincter proportions and that ANN regression can be used to clarify the complex coordination of sphincter muscles. Furthermore, the ensemble model might have taken notable advantage of the ANN performance, improving model performance. To prove this hypothesis, we plan to analyze whether the algorithm improves AUC in high‐resolution images focusing specifically on the periurethral lesion or levator ani muscles when other factors are eliminated as much as possible.

Our study was an evaluation of intraoperative images, not a study to “exclude unfit patients for surgery,” as focused on in previous studies using preoperative information.^7^ However, our results indicate that both “anatomical” and “surgical factors (surgical techniques)” shown in the video are related to the risk of PPUI. Our results suggest that the information obtained from actual surgical images is more important for outcome prediction than surgical procedure and performance records transcribed from surgical records and databases. Identifying the hotspots in the black box of DL can help clarify the mechanism of PPUI from multiple perspectives, which may contribute to preoperative informed consent (to explain the prediction of PPUI) and education, including the improvement of surgical techniques. The most significant aspect of this study is its clarification of this point. On the other hand, it is currently unclear whether intraoperative video imaging information encompasses preoperative MRI information. Thus, it may be necessary to use both preoperative MRI and intraoperative video images for analysis. Currently, it is difficult to extract valid features using CNN from two completely different dimensional pieces of information. However, such complex analysis may become possible by increasing the number of cases and improving CNN processing.

This study has several limitations. First, it was a retrospective study that was conducted at a single institute. Regarding this point, we obtained promising data as a result of internal validation. Further large multicenter studies are needed to externally validate our proposed algorithm. Second, PPUI is defined solely on the basis of daily pad use, even though no standard PPUI definition has been established. Third, it remains unclear if the current AI methods are reproducible for predicting long‐term UI and pad‐free continence, since our data has one time point for continence data; the high AUC continence observed at 3 months with 0–1 pad has not been reproduced in men without pads or noted at the 6‐ or 12‐month follow‐up. Fourth, external validation was not enforced and the objectivity of the data is not ensured at present.

## 5 CONCLUSION

Our findings may be useful for individual counseling within clinical practice, utilizing preoperative and intraoperative information to calculate the probability of UI after RARP surgery. It is expected that a method for identifying hotspots of intraoperative video information will be developed in the future, and that this DL model can be used as a tool for surgical navigation training to avoid prolonged UI after RARP surgery.

## AUTHOR CONTRIBUTIONS

**Wataru Nakamura:** Conceptualization (supporting); data curation (equal); formal analysis (supporting); investigation (supporting); methodology (supporting); resources (supporting); validation (supporting); writing – original draft (equal). **Makoto Sumitomo:** Conceptualization (lead); data curation (supporting); formal analysis (lead); investigation (lead); methodology (lead); project administration (lead); resources (supporting); software (supporting); supervision (equal); validation (equal); visualization (lead); writing – original draft (equal); writing – review and editing (equal). **Kenji Zennami:** Data curation (supporting); resources (supporting); validation (supporting). **Masashi Takenaka:** Data curation (supporting); resources (supporting); validation (supporting). **Manabu Ichino:** Data curation (supporting); resources (supporting). **Kiyoshi Takahara:** Conceptualization (supporting); data curation (supporting); methodology (supporting); resources (supporting). **Atsushi Teramoto:** Conceptualization (supporting); data curation (equal); formal analysis (equal); investigation (supporting); methodology (equal); software (equal); supervision (supporting); validation (equal); writing – original draft (supporting); writing – review and editing (supporting). **Ryoichi Shiroki:** Conceptualization (supporting); data curation (equal); methodology (supporting); project administration (supporting); resources (lead); supervision (lead).

## CONFLICT OF INTEREST STATEMENT

The authors have stated explicitly that there are no conflicts of interest in connection with this article.

## ETHICS STATEMENT

This study was approved by the Institutional Review Board (IRB) of Fujita Health University Hospital (IRB no. HM19‐257).

## Supporting information


**TABLE S1.** AUC information based on six methods.

## Data Availability

The data sets used and/or analyzed during the current study are available from the corresponding author upon reasonable request.
