# Supplementary material for: Combination of deep learning and ensemble machine learning using intraoperative video images strongly predicts recovery of urinary continence after robot‐assisted radical prostatectomy
Source: Cancer Rep (Hoboken). 2023 Jul 14;6(9):e1861. doi: 10.1002/cnr2.1861 (PMC10480482; doi:10.1002/cnr2.1861)
Supplement: Supplementary file 1 — TABLE S1. AUC information based on six methods. [file CNR2-6-e1861-s001.docx]

**Supplementary Table 1. AUC information based on six methods**

CA: classification accuracy

F1: 2 x Precision x Recall / (Precision + Recall)
